# Timing Matters: The Interplay between Early Mealtime, Circadian Rhythms, Gene Expression, Circadian Hormones, and Metabolism—A Narrative Review

**DOI:** 10.3390/clockssleep5030034

**Published:** 2023-09-06

**Authors:** Ahmed S. BaHammam, Abdulrouf Pirzada

**Affiliations:** 1 The University Sleep Disorders Center, Department of Medicine, College of Medicine, King Saud University, Riyadh 11324, Saudi Arabia; 2 North Cumbria Integrated Care (NCIC), National Health Service (NHS), Carlisle CA2 7HY, UK; roufi786@gmail.com

**Keywords:** dawn, melatonin, clock genes, metabolic health, glucose control, dinner, cortisol

## Abstract

Achieving synchronization between the central and peripheral body clocks is essential for ensuring optimal metabolic function. Meal timing is an emerging field of research that investigates the influence of eating patterns on our circadian rhythm, metabolism, and overall health. This narrative review examines the relationship between meal timing, circadian rhythm, clock genes, circadian hormones, and metabolic function. It analyzes the existing literature and experimental data to explore the connection between mealtime, circadian rhythms, and metabolic processes. The available evidence highlights the importance of aligning mealtime with the body’s natural rhythms to promote metabolic health and prevent metabolic disorders. Specifically, studies show that consuming meals later in the day is associated with an elevated prevalence of metabolic disorders, while early time-restricted eating, such as having an early breakfast and an earlier dinner, improves levels of glucose in the blood and substrate oxidation. Circadian hormones, including cortisol and melatonin, interact with mealtimes and play vital roles in regulating metabolic processes. Cortisol, aligned with dawn in diurnal mammals, activates energy reserves, stimulates appetite, influences clock gene expression, and synchronizes peripheral clocks. Consuming meals during periods of elevated melatonin levels, specifically during the circadian night, has been correlated with potential implications for glucose tolerance. Understanding the mechanisms of central and peripheral clock synchronization, including genetics, interactions with chronotype, sleep duration, and hormonal changes, provides valuable insights for optimizing dietary strategies and timing. This knowledge contributes to improved overall health and well-being by aligning mealtime with the body’s natural circadian rhythm.

## 1. Introduction

Metabolic disorders have a substantial impact on a large population worldwide, leading to decreased quality of life, increased healthcare utilization, and shortened life expectancy [1]. Numerous risk factors contribute to an increased susceptibility to metabolic diseases, necessitating a systematic approach to comprehend these factors. The timing of fasting/feeding, mealtimes, circadian rhythm, and sleep, as well as the complex interplay between these elements, may exert influence on the circadian rhythms of various organs and cells within the body, particularly when food intake occurs at inappropriate intervals relative to the body’s circadian clock [2].

In humans and mammals, the internal circadian timing system synchronizes with the 24-h light–dark cycle by receiving light signals that reach a central clock in the hypothalamus [3]. The circadian rhythm, an endogenous timekeeping system, is crucial in regulating various physiological processes in the human body. This biological clock is responsible for maintaining a 24-h cycle that influences the timing of sleep, metabolism, body temperature, and hormone secretion, and the internal clock was for centuries synchronized with mealtimes at specific periods. Moreover, the circadian rhythm and meal timing mutually interact; virtually all mouse models with diet-induced dysmetabolism exhibit disrupted feeding patterns, frequently marked by the absence of specific mealtimes and the dispersion of caloric consumption across both day and night [4].

The widespread availability of electrical lighting has increased nocturnal activities and granted humans the ability to personally select their light–dark cycles and prolong wakefulness activities well into the night [5]. This ability to modify the timing of wakefulness can lead to a misalignment between the external (environmental) and internal circadian physiology [6]. Disharmony in circadian rhythms can compromise the functioning of these organs and impact the overall metabolic systems in the body [7] and has been correlated with adverse health outcomes, including diminished energy metabolism [8], impaired glucose metabolism [9], and heightened risk of cardiovascular disease [10].

Furthermore, consuming meals during nighttime hours can disrupt sleep latency, duration, and quality [7]. Insufficient sleep duration and poor sleep quality are established risk factors for metabolic diseases [7]. Additionally, emerging evidence has indicated a connection between eating patterns, mealtimes, and metabolic health in humans [11]. Current evidence from animal studies [12] and human research [13] suggests that consuming food during periods typically allocated for sleep can lead to increased weight and detrimental metabolic health. Groups prone to shifting their activities to later hours, like night or rotating shift workers, or some young people, such as teenagers and college students who stay awake at night, also exhibit a higher prevalence of weight gain and obesity [14]. Numerous circadian events occurring at dawn have been observed in humans, including changes in blood glucose, insulin sensitivity, and hormone levels (such as cortisol), as well as heightened activity in the autonomic nervous system [15,16], which could be linked to early morning meals.

Therefore, this narrative review provides an overview of recent research exploring the importance of synchronizing central and peripheral body clocks and meal timings and the interactions with gene expression, circadian hormones, and metabolism. It focuses on data looking at changes during the early hours of the morning (dawn) and evening (dusk) and their influence on metabolism, with a specific focus on the role of early mealtimes.

## 2. Search Methods

We adhered to established criteria for searching and reporting the literature for narrative reviews [17,18,19]. The PubMed database and Google Scholar were utilized to conduct the search using keywords such as “mealtime”, “meal timing”, “eating schedule”, “early meal”, “delayed meal”, “circadian rhythm”, “breakfast”, “dinner”, “circadian clock”, “biological clock”, “chrononutrition”, “circadian misalignment”, “metabolism”, “metabolic rate”, “energy expenditure”, “obesity”, “body mass index”, “adiposity”, “weight”, “appetite”, “cortisol”, “melatonin”, “insulin”, “clock genes”, and “cardiovascular health”.

Inclusion criteria encompassed original articles (human and animal studies) and systematic reviews published up to 31 May 2023, with no age restrictions. Exclusion criteria included editorials, opinions, and articles written in languages other than English. To identify additional relevant publications, the reference lists of retrieved articles were reviewed (backward search), and recent articles citing the retrieved papers were examined (forward search). The initial search identified 1598 papers of which 139 articles met the inclusion criteria.

For studies relevant to this review’s objectives, information about study design, development, and interventions was extracted. Data collection focused on intervention type, goals, target population, study design and conduct, findings, participant demographics, intervention duration, and results. The investigators’ conclusions were also noted. Both authors of this review independently assessed the retrieved papers for eligibility and extracted study data. Any discrepancies were resolved with discussion and agreement. During data analysis, tables were created by both authors to describe the studies and summarize the results, ensuring accuracy and comprehensiveness.

## 3. A Brief History of Meal Timings

Meal timing has witnessed changes in different cultures over time [20,21,22,23]. Moreover, religion may have an impact as well on mealtimes. The timing of meals during the Middle Ages was shaped by the presence of daylight. In a time without electricity, people would rise earlier to utilize natural light for a range of tasks, including meal preparation and eating [24]. Due to the absence of electricity, the option to cook dinner in the evening was not available. Peasants had their main meal around midday, although it was a considerably simpler and less extravagant event [24]. Ancient traditional Chinese Medicine, practiced for over 2500 years, suggests that the ideal time for carbohydrate-rich meals is between 0700 and 1100, with smaller meals recommended later in the day during the transition from an active phase (“yang”) to a resting phase (“yin”) [20,25]. This practice is rooted in the belief that consuming energy-dense meals in the evening can disrupt sleep and various bodily functions. In medieval England, dinner, the main meal of the day, was consumed around noon or 01:00 p.m., while supper, a smaller meal, was closer to sunset, typically between 04:00 p.m. and 06:00 p.m. [20,26]. Religion also impacts mealtime; for example, during Islamic fasting inside or outside the month of Ramadan, followers refrain from eating and drinking between dawn and sunset for a month and are requested to eat an early pre-dawn meal called Suhur before fasting, which has a religious dimension [27]. Also, during ordinary days, Muslims were historically required to wake up every day for the dawn prayer, where they eat a meal after the prayer and start their day [27]. The advent of artificial lighting, such as oil lamps, led to a shift in dinner and other activities to later times. By the late eighteenth century, dinner had moved to around 04:00 p.m. or 05:00 p.m., and the introduction of lunch was a response to extended fasting periods between breakfast and dinner. The Industrial Revolution also contributed to later dinner times among working-class men who split the workday with a quick noon meal [20,26]. In the USA, an examination of data derived from the National Health and Nutrition Examination Survey (NHANES) demonstrated that snacks consumed after dinner constituted the largest share of calories, providing nearly 45% of overall calorie consumption [28]. Over 40 years, from 1971–1974 to 2007–2010, the timing of breakfast and lunch generally shifted to later times, while dinner timing remained stable [28]. In the most recent NHANES analysis involving 15,341 adults from the 2009 to 2014 cycle, the average dinner time was 6:24 p.m., with the average time of the last eating episode being 8:18 p.m. [28]. In fact, contrary to the commonly held belief that humans follow a strict three-meal pattern, it is currently evident that calorie intake occurs sporadically and over an extensive timeframe throughout the 24-h cycle [29]. Hence, limiting food intake to predetermined time windows may present a readily implementable behavioral intervention with the potential to enhance outcomes in patients with metabolic syndrome.

## 4. Regulation and Control of the Circadian Body Clocks

Every organ, tissue, and cell in the body functions according to a biological clock that adheres to a circadian rhythm. Biological clocks can be classified into central and peripheral clocks depending on where they are located anatomically [30]. The primary biological clock is situated within the hypothalamus, precisely within the suprachiasmatic nucleus (SCN), while peripheral clocks exist in all cells throughout the body [31]. These clocks help maintain normal tissue function by regulating the activity of tissue-specific genes [31]. The body’s internal clocks, known as circadian rhythms, rely on independent oscillators within cells to regulate gene expression; circadian clocks are intrinsic biological oscillators that exhibit a periodicity of approximately 24 h [32], even without external cues [33]. To achieve external and internal synchrony, the body’s biological clocks must be entrained daily.

Circadian clocks are intrinsic biological oscillators that exhibit a periodicity of approximately 24 h [32]. Found in a wide range of light-sensitive organisms, these clocks play a vital role in coordinating rhythmic activities in accordance with the natural daily cycles [34]. In order to stay synchronized with the external environment, circadian clocks rely on external signals called zeitgebers or timing cues to undergo phase resetting [32,34]. The retinohypothalamic tract primarily entrains the SCN through exposure to external light, whereas neurohormonal factors exert an influence on peripheral clocks and mealtimes [35,36], and peripheral tissues can be moderately affected by nonphotic signals, such as food intake and glucocorticoids [37,38]. The nervous and endocrine systems both regulate the peripheral clocks in vivo. One example is the secretion pattern of glucocorticoids, which is regulated by the SCN and helps synchronize peripheral clocks [38,39]. Exposure to intense light during nighttime can disrupt the secondary clocks and rhythms that are controlled by the central clock situated in the SCN; similarly, eating meals during the night can also disrupt the peripheral clocks.

External or internal circadian system desynchrony has been associated with diverse metabolic dysfunctions, including compromised glucose tolerance and diminished insulin sensitivity, elevated susceptibility to complications related to reduced insulin sensitivity, such as non-alcoholic fatty liver disease (NAFLD), heightened levels of proinflammatory cytokines, elevated arterial blood pressure, and decreased energy expenditure, leading to obesity [40,41,42]. Moreover, epidemiological data support the connection between circadian misalignment and elevated susceptibility to metabolic disorders, diabetes, disorders affecting the cardiovascular system, and NAFLD [43,44,45,46].

Laboratory-controlled studies suggest that acute circadian rhythm misalignment increases the risk of developing metabolic disorders [11]. However, more research is needed in the general population as well as long-term studies to investigate the potential for adaptation to long-term disruption of the circadian rhythm [47].

## 5. Clock Genes and Circadian Rhythms

Clock genes are central to circadian rhythms, profoundly influencing behavior and human physiology [48]. These genes, discovered in fruit flies and mammals like mice, form networks that display a 24-h oscillation [49,50]. These networks not only govern physiological and behavioral rhythms but extend their influence to other cellular functions [51]. Mammalian circadian rhythms arise from a feedback loop involving transcriptional activators and repressors [52]. Key proteins, BMAL1 and CLOCK, stimulate the transcription of Cry and Per genes. When repressor proteins CRY and PER reach a specific concentration, they interfere with the CLOCK-BMAL1, starting a new transcription cycle [33]. Current studies underline the significance of circadian rhythm in health disorders and its synchronization for optimal cellular functioning [53]. Moreover, there is a documented interplay between clock genes and feeding times, with feeding rapidly impacting circadian clock gene expression in animals [54,55]. For nocturnal rodents, daylight feeding alters circadian clock phases in peripheral tissues but not in the central timekeeper [56]. The relationship between human feeding times and clocks is an emerging field, suggesting that timed feeding could have clinical benefits [57].

## 6. Mealtime and Cardiometabolic Risk

Emerging evidence highlights the significant role of mealtime in controlling metabolic processes and its close interaction with the biological clock [11,58]. Chrononutrition, a new discipline, addresses the interplay between mealtime, circadian rhythm, and metabolic regulation [59]. Current research indicates that meal timing influences the circadian cycle, metabolic regulation, and body weight [60]. Consuming meals during inappropriate time periods can cause misalignment between peripheral biological clocks and the central biological clock in the SCN, increasing the potential for developing metabolic disorders [7,36,58]. Studies in both nocturnal species and humans have shown comparable results, wherein consuming meals at inappropriate hours (dark hours, “inactive phase” for humans) is linked to an increased likelihood of experiencing metabolic impairment [61]. In shift workers, night eating has been linked to metabolic disturbances [62,63]. Research has also shown that confining the timing of food intake to either daytime or nighttime can influence the risk of metabolic disorders [29,64,65,66]. However, it is essential to acknowledge that studying the intricate interplay between mealtime, circadian rhythm, and metabolism poses challenges due to the influence of multiple interacting factors. Factors including individual chronotype, lifestyle elements like shift work, sleep disruptions and sleep patterns, dietary composition and portion sizes, physical activity levels, environmental factors including nighttime light exposure, age, and genetic factors can interact with these rhythms, with some evidence suggesting that high-fat meals eaten late at night may be particularly disruptive [58,67,68].

In addition to caloric intake control, maintaining a consistent meal schedule is crucial as it aids in the effective management of energy balance. Consuming meals at set times from sunrise to sunset (between dawn and dusk rather than continuously throughout the day) enhances circadian rhythmicity and promotes a beneficial cycle for optimal metabolic health [20]. In experiments conducted on Wistar rats, it was observed that providing a daily serving of chocolate during the period of activity (breakfast) facilitated the adjustment of the SCN activity to the new schedule in a model of jet lag, leading to faster re-entrainment [69]. Additionally, in a model using rats simulating shift work, having a daily serving of chocolate coinciding with the start of the active phase (breakfast time) prevented disruption of the body’s internal clock by increasing the strength of the day–night activation in the SCN, specifically involving c-Fos (c-Fos is a helpful physiological marker of neural activation, which proved to be successfully utilized in the SCN) [69,70]. In contrast, chocolate consumed during dinner hindered re-synchronization in the jet lag condition and fostered disruption of circadian coordination in shift work models [69]. Furthermore, rats consuming chocolate during breakfast showed lower weight gain, while those having chocolate during dinner exhibited increased body weight [69]. These findings emphasize the importance of meal timing in regulating circadian synchrony and metabolic function, particularly for a high-calorie and appetizing meal like chocolate.

The favorable impacts of morning meal (breakfast) consumption on body weight and cardiometabolic well-being have not been consistently reported in all studies [71,72]. A consensus statement from the American Heart Association concluded that while epidemiological evidence indicates a potential adverse impact of consuming meals late in the day on cardiometabolic hazard, the scope of clinical intervention studies addressing this issue has been scant and lacks the specific focus to draw definitive conclusions or formulate recommendations [36]. The statement also emphasized that mealtime and frequency are not the sole determining factors; the length of time between meals and caloric intake are also crucial considerations [36]. In the following sections, we explore the literature that examines the effects of both early and late mealtimes. By thoroughly examining these studies, we aim to gain comprehensive insights into the implications and outcomes associated with different mealtime choices.

## 7. Dawn and Dusk Feeding Time: The Transition from Fasting to Feeding and Feeding to Fasting

### 7.1. Time of the Day and Clock Genes

To comprehend breakfast and dinner timings, it is essential to understand the physiological changes occurring in the early morning. These changes offer insights into the factors influencing meal timing and overall health. Clock genes, which regulate circadian rhythms, may be impacted by the timing of dawn and dusk [73]. At dawn, the central clock in the SCN, activated by increasing light, signals the transition to daytime and feeding states, crucial for regulating glucose metabolism [74]. This regulatory system involves a complex interaction of genes, such as CLOCK-BMAL1, PER, and CRY, with feedback cycles that adapt to morning light levels and evening factors [75,76,77].

Our daily feeding rhythm, like the sleep–wake cycle, emerges from homeostatic needs and temporal controls. Typically, humans consume two to three meals daily, regardless of external cues. Yet, meal timings can vary across individuals or cultures, possibly interacting with clock genes. Notably, rats show that fasting and refeeding patterns align with their circadian rhythm, emphasizing the connection between meal timing and the circadian clock [78].

Research indicates that early breakfast activates the “CLOCK: BMAL1” complex, initiating gene transcription of PERs and CRYs, and subsequent physiological processes [2,79]. Interactions between genes such as AMPK, SIRT1, and CLOCK-BMAL1 drive tissue-specific expressions like increased insulin secretion, enhanced GLP-1 response post meals, and glucose uptake in muscles [80]. At night, clock genes promote hepatic glucose production and other metabolic activities. Therefore, feeding around dawn is pivotal for activating the molecular clock and daily physiological regulation [80].

### 7.2. Early Morning Meal

Interestingly, ghrelin, a hormone that stimulates appetite and food intake, reaches its peak secretion at 08:00 in the morning [81]. Likewise, adiponectin levels experience a morning peak around 11:00 a.m. before declining by 08:00 p.m. [82]. The morning surge of adiponectin activates AMPK (AMP-activated protein kinase), enhances the oxidation of fatty acids, improves insulin sensitivity, increases glucose uptake in muscles, and stimulates glycolysis [82]. This leads to a reduction in hepatic glucose production, resulting in increased glucose utilization and decreased fat accumulation during the early hours of the day [14,83]. In contrast, lower levels of adiponectin in the evening bring about metabolic changes that favor insulin-mediated anabolic processes [2]. As a result, the insulin response following a meal during the evening promotes the accumulation of fat and the process of lipogenesis through the activation of fatty acid synthesis [14,82]. Conversely, elevation in the leptin hormone (a hormone associated with satiety) in the evening reduces fat accumulation while increasing nocturnal lipolysis. Therefore, having breakfast at an earlier time (dawn meal) aligns with the transition of the circadian clock, facilitating metabolic processes like glucose uptake, insulin sensitivity, and the production of glycogen. This synchronization of mealtime with the central clock aids in maintaining the coordination of peripheral clocks in metabolic tissues such as the liver, skeletal muscle tissue, and fat tissue [2].

Also, the consumption of breakfast at dawn upregulates the expression of clock genes, such as CLOCK, BMAL1, and RORα, which are involved in regulating insulin sensitivity, glucose uptake, and energy expenditure [84]. This upregulation in clock genes also influences the secretion of hormones like GLP-1, which is elevated following meals consumed during the early part of the day in comparison with isocaloric meals consumed in the evening [85]. Thus, having an early morning breakfast is essential for maintaining the synchronization of central and peripheral clocks and promoting metabolic homeostasis. Moreover, breakfast also appears to be important for having a good night’s sleep, which in turn can contribute to earlier bedtimes and rise times and promote a healthier circadian rhythm. A recent exploratory study examined the correlation between breakfast consumption and subjective sleep quality among university students [86]. The findings indicated that students who skipped breakfast, consumed late-night snacks, or substituted meals with snacks were more prone to experiencing overall poor sleep quality [86]. A subsequent, more recent study investigated the relationship between eating habits and sleep difficulties in a large sample of children and adolescents [87]. It revealed that more frequent breakfast consumption and higher intake of fruits and vegetables were linked to fewer sleep difficulties [87].

The regulation of clock genes has been found to improve insulin sensitivity, the responsiveness of β-cells, the activity of GLUT-4, glucose uptake in muscles, and the secretion of post-meal incretins (GLP-1, GLP-2, and GIP); the ability to stimulate insulin release is mediated by incretins during the early hours of the active phase [2]. Various studies have supported this; consequently, various studies have reported that consuming identical meals in the evening leads to a considerably greater glycemic response compared with the morning, indicating a notable disparity in how the body processes and responds to the same meals based on the timing of consumption [85,88,89,90,91,92,93,94]. Table 1 presents a summary of randomized controlled trials that assessed the relationship between mealtimes, circadian rhythm, and metabolism [65,95,96,97,98,99,100,101,102,103,104,105,106].

### 7.3. Energy Expenditure and Circadian Rhythm

The energy expenditure at rest (resting energy expenditure or REE) follows a pattern of fluctuations throughout the day, with the lowest levels occurring during the resting phase [110]. In contrast, the respiratory quotient (RQ), which indicates the utilization of macronutrients, attains its highest level during the early part of the active phase [110]. Moreover, it has been suggested that the thermic effect of food, which refers to the increase in the utilization of energy after eating, is lower in the evening compared with the morning. It seems that the body’s internal clock boosts the thermogenic response to food (diet-induced thermogenesis or DIT) following meals consumed during the early part of the active phase, particularly breakfast, in comparison with the evening [111,112,113,114]. This variation in thermogenesis may be attributed to the influence of circadian rhythms [111]. Furthermore, endocrine factors in the human body may reach their peak at different times of the day due to natural oscillations [115,116,117]. For instance, during the active phase in the morning (around 7 a.m. to 8 a.m.), there is a peak in the hormone cortisol, which plays a role in regulating energy levels and preparing the body for activity [118,119].

In a recent study utilizing a randomized crossover design, the researchers compared early eating (between 08:00 a.m. and 06:00 p.m.) to isocaloric late eating (between 01:00 p.m. and 11:00 p.m.) in overweight and obese adults; it was observed that late eating led to higher levels of hunger upon waking, reduced energy expenditure, and decreased serum leptin while increasing the ghrelin-leptin ratio [107]. Another noteworthy finding was that late eating influenced the expression of genes related to adipose tissue, favoring lipid storage by downregulating genes associated with the MAPK (p38 mitogen-activated protein kinase) pathway, the TGF-β signaling pathway, modulation of insulin receptor tyrosine kinases, and autophagy. These changes ultimately promoted increased adipogenesis and may contribute to an elevated risk of obesity in humans [107]. These results are in line with previous findings demonstrating that consuming a diet mainly during the morning hours significantly reduces hunger scores [2,84,120,121].

Analysis of gene expression in adipose tissue while abstaining from food until noon revealed changes in lipid metabolism pathways. These changes included alterations in the signaling pathway of transforming growth factor-beta (TGF-β) and the regulation of receptor tyrosine kinases, which were consistent with an increment in adipogenesis and a reduction in lipolysis [107]. Moreover, within the dietary intervention protocol that involved consuming meals later in the day, fasting until 01:00 p.m. resulted in a notable reduction in energy expenditure when compared with the dietary intervention protocol involving early meal consumption that included an early timed breakfast [107]. The observed reduction in the amount of energy expended during the fasting period until noon suggests that the arrangement of omitting breakfast could potentially elevate the likelihood of developing obesity and diminish the efficiency of strategies used for weight loss [2]. Conversely, consuming a high-energy breakfast early in the day may aid in weight loss by suppressing appetite [2]. Additionally, this provides a detailed explanation of the underlying mechanisms for the improved results in terms of weight loss observed when more energy is moved to the early part of the day in the DI [122]. Hence, the early hours of the active phase, particularly breakfast, are the ideal time for consuming food, especially for consuming carbohydrates. In contrast, it may be more suitable to reduce energy and carbohydrate intake during the evening and nighttime [123,124,125,126].

### 7.4. Mealtime, Insulin Sensitivity, and Glucose Response

Consuming breakfast early in the day has a positive impact on the interaction between SIRT1 and AMPK, leading to improved insulin sensitivity, translocation of GLUT-4, and uptake of glucose by muscles. This, in turn, results in better glucose and insulin responses after a meal in the morning compared with the evening. The increased expression of CLOCK and PER2 genes triggers the activation of glycogen synthase 2 (GYS2) gene transcription and promotes the synthesis of glycogen in the liver. As a result, excess glucose derived from the meal is conveyed to the liver and stored as glycogen, whilst the breakdown of glycogen (glycogenolysis) is restricted. Achieving metabolic homeostasis may depend on the temporal alignment of breakfast with the presence of light at dawn.

A study was carried out to examine the impact of prolonged fasting until noon versus eating an early breakfast on the expression of clock genes and the response of glycemic, insulin, and incretin levels [127]. The study included both healthy individuals and individuals diagnosed with type 2 diabetes (T2D) [127]. Participants were randomly assigned to two single-test days: one involving consuming breakfast early in the morning at 8:00 a.m., followed by lunch and dinner, and the other day with solitary lunch and dinner meals, omitting breakfast [127]. Extended fasting for approximately 16 h until noon on the no-breakfast day altered the expression of clock genes and decreased the mRNA expression of AMPK, BMAL1, PER1, and RORα before and after lunch. This fasting regimen was linked to elevated glucose levels and impaired and delayed release of insulin, while intact GLP-1 responses after lunch were reduced compared with the breakfast day. In contrast, breaking the overnight fast with a high-energy breakfast at 8:00 a.m. on breakfast day exerted a resetting influence on the mRNA expression of these pivotal metabolic clock genes [127]. As a result, there was a notable decrease in postprandial blood sugar levels and enhanced and expedited insulin and GLP-1 responses following the midday meal (lunch) [127].

The findings indicate that following a daytime eating schedule may be beneficial for weight loss and metabolic improvements in real-world conditions. The improved metabolic effects linked to consuming breakfast may be attributed to food serving as a regulator of the peripheral clocks, potentially aligning, and coordinating their functions. However, further large-scale randomized controlled trials are needed to thoroughly evaluate the effects of breakfast consumption on weight and cardiometabolic risk.

### 7.5. Early Breakfast or No Early Breakfast

The definition proposed by Timlin and Pereira [128] is widely recognized as the academic standard. According to their definition, breakfast is the initial meal of the day, ingested either before or at the onset of daily activities such as duties, travel, or work. This meal should be eaten within two hours of waking up, preferably no later than 10:00 a.m. in the morning, and should provide an energy intake ranging from 20% to 35% of the individual’s total daily energy requirements. However, it is crucial to acknowledge that this definition does not specify the duration of the overnight fast, which is relevant in the present discussion. Moreover, there is inconsistency in the interpretation of breakfast across various research studies, and there is no unanimous agreement regarding the positive impact of consuming breakfast on weight and cardiometabolic health, as reported in various studies.

A recent meta-analysis comprising 12 randomized controlled trials, sourced from affluent countries, aimed to evaluate the influence of consistent breakfast consumption on body weight and energy consumption in adults [129]. The results of this analysis revealed a slight disparity in weight, with a preference toward individuals who skipped breakfast (mean follow-up of 7 weeks; range of 2–16 weeks). However, the results across trials were inconsistent, and individuals assigned to the breakfast group exhibited elevated levels of daily energy intake (approximately 260 extra calories/day) in comparison with individuals who did not consume breakfast (mean difference of 259.79 kcal/day). Moreover, overall, the studies included in the analysis generally exhibited low methodological quality, leading the authors to caution against definitive interpretations of their findings [129].

In the majority of breakfast-related studies that compared the impacts of having or skipping breakfast, there was a lack of consideration for the length of the overnight fasting period [130]. This factor, which could significantly influence metabolic outcomes, was an important variable that should have been considered. To illustrate, let us consider two individuals who both had their last meals at 11:00 p.m. the previous night. One of them had breakfast at 05:00 a.m. the following morning, while the other consumed breakfast at 11:00 a.m. The discrepancy in the duration of their overnight fasting periods could potentially be the reason behind notable metabolic differences [131]. Conversely, if individuals had similar durations of overnight fasting, regardless of the timing of their meals, it is possible that they would exhibit similar metabolic profiles.

Another more recent comprehensive evaluation and synthesis of observational studies indicated that skipping breakfast in real-world settings may contribute to weight gain and the onset of excess weight and obesity [132]. However, these findings should be interpreted cautiously due to the limited number of studies and the heterogeneity observed, which may introduce publication bias and a small study effect. The methodological differences among the included studies and the lack of information on breakfast composition, quantity, and quality limit the precision and robustness of the results.

These inconsistencies highlight the need for large, high-quality, randomized controlled trials to comprehensively evaluate the influence of breakfast consumption on body weight and the risk of cardiometabolic conditions. A new consensus statement by the American Heart Association acknowledged that data from population-based studies indicate a potentially harmful effect of late mealtime on cardiometabolic risk. Nevertheless, conducting intervention studies that establish a causal relationship and provide conclusive evidence, along with specific recommendations, has been challenging due to limitations in focus and methodology [36]. Furthermore, the consensus statement emphasized that mealtime and frequency alone are not the sole factors contributing to these outcomes; the duration between meals and the caloric intake in each meal are also important considerations [36]. Studies investigating the impact of Ramadan daytime fasting, where individuals consume breakfast in the early morning (30 min before dawn) and late evening meals (at sunset), have shown reductions in weight and cardiometabolic risk [7,133]. It is important to note that observational studies have limitations in that they can only establish associations between behaviors and diseases, without being able to ascertain causality or directionality.

A recent small, randomized crossover trial of healthy adult participants suggested that disruption of circadian rhythms induced by delaying mealtimes by 4 h relative to circadian alignment shifts nutrient metabolism, leading to elevated carbohydrate oxidation and decreased fat oxidation [134]. Despite these metabolic changes, the 24 h energy expenditure remained consistent between the two conditions. In another controlled randomized experimental crossover study conducted in free-living conditions, eight young and healthy lean volunteers followed two-week and eight-week eating schedules [135]. The schedules, a daytime schedule (08:00 a.m.–07:00 p.m.) and a delayed schedule (12:00 p.m.–11:00 p.m.), were counterbalanced with a 2-week washout period in between [135]. The results showed that the daytime schedule led to weight loss, improved energy metabolism, reduced insulin resistance, and favorable changes in glucose and lipid profiles compared with the delayed schedule [135]. These findings suggest that following a daytime eating schedule can be an effective and feasible behavioral modification to promote weight loss and metabolic improvements in real-world conditions.

These inconsistencies highlight the need for large, high-quality, randomized controlled trials to comprehensively assess the impact of breakfast consumption on weight and cardiometabolic risk.

### 7.6. Skipping Breakfast and Genetics

In addition to the above, a group of scholars developed a genome-wide association study (GWAS) on breakfast skipping using the UK Biobank dataset, which consisted of approximately 200,000 participants [122]. The results were then replicated in other European populations, including Twin UK and CHARGE [136]. The study identified six genetic variants associated with breakfast skipping. These variants were found to be involved in caffeine metabolism, carbohydrate metabolism, and the control of the biological rhythm. The findings from this large-scale study, involving 200,000 participants, provided evidence suggesting a causal association between genetically determined breakfast skipping and obesity.

A Mendelian randomization analysis provided evidence suggesting a causal relationship between skipping breakfast and obesity [137]. However, it is important to interpret these results with caution, considering the limitations associated with Mendelian randomization. One such limitation is that DNA alone does not encompass all the necessary information to fully determine a phenotype, among other potential limitations [136].

### 7.7. Evening and Late-Night Meals

At dusk, the central clock in the SCN responds to the decrease in light, signaling the transition from the daytime feeding state to the nocturnal fasting state. In simple terms, eating late at night and going to bed soon after a late-night dinner can cause a prolonged elevation in blood glucose levels after eating, especially if there is an absence of physical activity during sleep. This can make it harder for blood sugar levels to return to normal when breakfast is eaten without enough time in between. While it may be helpful for most people to eat breakfast, those who regularly eat dinner late at night and go to bed soon after should consider their late-night dinner habits and overall lifestyle when deciding whether to eat breakfast to improve their heart and metabolic health [138].

In theory, having dinner late at night, especially right before going to bed, can cause a long-lasting increase in blood sugar levels after eating. This is because of various influences, like the lack of physical activity during sleep [139]. A cohort study was the first to provide evidence of the connection between consuming dinner late at night and blood sugar control in individuals with type 2 diabetes [140]. It found that having dinner after 8 p.m. was independently associated with an increase in HbA1c [140]. In several short-term trials, both healthy individuals and those with type 2 diabetes showed higher levels of blood glucose and insulin after eating meals at night [141]. These studies suggest that disrupting the body’s natural circadian rhythm can worsen the normal decrease in glucose tolerance that occurs at night. Moreover, findings derived from the KNHANES study indicated a correlation between eating late at night and a higher prevalence of metabolic syndrome [142]. Also, late-night eating was linked to a reduction in high-density cholesterol.

Consuming meals late in the day can potentially disrupt the synchronization between the central and peripheral clocks, potentially contributing to the emergence of metabolic disorders [36]. At the same time, new data suggest that consuming an early dinner could be advantageous in maintaining a modest postprandial glycemic response and stable blood glucose levels during the nighttime [100,143]. A randomized controlled trial study examined the effects of early time-restricted eating on blood glucose levels and postprandial lipid metabolism in healthy adults. The results showed that eating dinner at 06:00 p.m., as opposed to 09:00 p.m., led to improved 24 h blood glucose levels and better substrate oxidation after breakfast on the following day [100]. Another randomized crossover trial explored the effects of excessive consumption of a late dinner on nocturnal metabolism in young, healthy volunteers (*n* = 20) [144]. The study found that eating a late dinner (at 10:00 p.m. vs. 06:00 p.m.) had higher glucose levels, delayed triglyceride peak, reduced free fatty acids, and dietary fatty acid oxidation; it also increased plasma cortisol levels [144]. These metabolic changes were most significant in individuals who typically went to bed earlier, as determined using actigraphy monitoring. These findings suggest that late dinners may contribute to nocturnal glucose intolerance and hinder fat metabolism, potentially promoting obesity if this pattern persists chronically.

However, some previous studies on time-restricted eating (TRE) may have been influenced by the inherent calorie restriction, making it difficult to separate the effects of TRE from calorie restriction [145]. A population-based study utilizing a larger population of the NHANES attempted to address this limitation by investigating the connection between fasting and cardiometabolic markers without the confounding factor of overlapping calorie restriction [145]. The data analysis revealed that delayed timing of the first meal was correlated with elevated levels of CRP, HbA1c%, insulin, glucose, total cholesterol, and LDL cholesterol. It was also associated with lower HDL cholesterol levels [145]. These findings suggest that initiating energy consumption earlier in the day can benefit cardiometabolic endpoints.

Late-night meals, particularly before bedtime, can contribute to metabolic disorders by elevating blood glucose levels and disrupting the body’s natural circadian rhythm. Conversely, consuming an early dinner has been associated with improved blood sugar control and better metabolic health. However, further research is needed to fully comprehend the overall impact of meal timing on health and to differentiate the effects of time-restricted eating from calorie restriction.

## 8. The Interaction between Mealtime and Circadian Hormones

The classical rodent circadian system simulation proposed by Pittendrigh and Daan (1976) [146] is also suggested to apply to humans. This model, along with its expansion by Illnerova and Vanecek (1982) [147], describes two distinct rhythmic components in the circadian timing system. One oscillator is aligned with dusk, regulating evening movement patterns and the onset of melatonin production in nocturnal rodents. The second oscillator is aligned with dawn, controlling morning locomotor activity and the cessation of melatonin secretion.

In a human study, clear patterns of wakefulness, internal body temperature, and hormone release were observed throughout the 24-h cycle [148]. These patterns demonstrate distinct diurnal and nocturnal states, with noticeable transitions resembling biological “dawn” and “dusk” [148].

In humans, the complex circadian pacemaker components synchronized with dusk and dawn play a role in regulating transitions in hormone secretion, such as melatonin and cortisol, during the evening and morning. These components also help adjust the timing of these transitions based on seasonal variations in daylight duration. Hormones like melatonin and glucocorticoids, tightly regulated by the master clock in the suprachiasmatic nucleus (SCN), influence the timing of secondary clocks expressing their respective receptors [149]. Peripheral clocks, even without a functional master clock, can maintain a daily rhythm under a light–dark cycle [150].

### 8.1. Cortisol

The circadian pattern of corticosteroids in diurnal and nocturnal mammals follows opposite patterns in accordance with the cycle of light and darkness, specifically aligning with dawn and dusk, respectively. However, it functions in both groups to anticipate the beginning of the daily period of wakefulness and activity, aiding in the activation of energy reserves and the stimulation of appetite [151,152]. Glucocorticoids play a crucial role as essential timing signals for numerous peripheral oscillators, facilitating their appropriate synchronization and adjustment to the light–dark (LD) cycle [38,153,154]. Furthermore, it has been suggested that they contribute to preventing sudden shifts in the timing of peripheral clocks within the circadian rhythm. This is particularly important when peripheral clocks become disconnected as a result of consecutive days of fasting and subsequent refeeding cycles [155]. Research has shown that individuals who fast from dawn to dusk exhibit two peaks (acrophases) of cortisol during dawn and dusk, compared with those who do not fast and individuals with a single peak (acrophase) [156]. These findings suggest that when meals are timed immediately before and after a fasting period that spans from dawn to dusk, the fasting-induced biphasic cortisol circadian rhythm synchronizes the peripheral clocks with the central clock, ensuring their phase alignment and thereby preventing phase shifts between the central and peripheral clocks.

Extensive research has focused on investigating the role of glucocorticoids, which have been linked to the central clock situated in the SCN [157,158]. The secretion of adrenocorticotropin hormone (ATCH) from the anterior pituitary gland is regulated by the SCN [159]. Consequently, this endocrine hormone plays a pivotal role in modulating the release of glucocorticoid hormones from the adrenal glands, which subsequently coordinate the functioning of peripheral tissue circadian clocks.

Studies have provided evidence showing that the regulation of clock gene expression and dietary rhythmicity involves the influence of glucocorticoid receptors [38]. Therefore, considering this relationship, it is plausible to suggest a potential connection between clock genes, the hypothalamic–pituitary–adrenal (HPA) axis, and metabolism [160].

Glucocorticoids, through the glucocorticoid receptor (GR), have been observed to exert a broad impact on gene expression [161]. Upon activation, the inactive complexed state of cytoplasmic GR experiences structural modifications and moves into the nucleus after dimerization. In the nucleus, the binding of GR to glucocorticoid response elements (GREs) facilitates the transcription of genes targeted by glucocorticoids [162]. These GREs are responsible for regulating the expression of several genes, including core clock genes such as Per1, Per2, Npas2, and Rev-erbß [163,164]. Both in laboratory settings (in vitro) and living organisms (in vivo), glucocorticoids have shown the capability to alter the circadian rhythms of peripheral clocks [38]. The synthetic glucocorticoid analog dexamethasone, for example, has been observed to stimulate the expression of clock genes and genes influenced by the circadian clock in rat fibroblasts [38]. Additionally, depending on the timing of administration to mice, dexamethasone was observed to either postpone or accelerate the timing of clock gene expression in the liver, kidney, and heart [38]. More recently, there is evidence suggesting that glucocorticoids can influence the human adipose tissue’s biological rhythms as well [165].

The association between meal timing and diurnal fluctuations in cortisol levels has been documented, highlighting the possibility of both adrenal and extra-adrenal regulatory influences [166]. Glucocorticoid levels are affected by meals and mealtime; it is suggested that when meals are timed immediately before and after dawn and dusk, the dual-phase cortisol circadian rhythm during periods of fasting aligns the timing of peripheral clocks with the central clock, promoting synchronization and preventing disruptions or transitions between the central and peripheral clocks [167].

In summary, the circadian pattern of corticosteroids aligns with dawn and dusk, following opposite patterns in diurnal and nocturnal mammals. Further research is needed to understand the impact of meal timing on cortisol regulation and metabolic health. Comprehensive multi-omics analyses in randomized-controlled trials can provide valuable insights into this relationship. Studies need to encompass circadian gene expression profiling, metabolomics, and proteomics to investigate the impact of meals around dawn and dusk in individuals suffering from chronic metabolic conditions and metabolic syndrome.

### 8.2. Melatonin

Melatonin levels follow a circadian pattern, reaching their highest point during sleep, decreasing toward the early morning hours, and remaining low until nighttime [168]. At night, melatonin levels begin to rise again in preparation for sleep. Apart from its function in regulating the sleep–wake cycle, melatonin also possesses antioxidant and anti-inflammatory properties and plays a role in controlling glucose and lipid metabolism and the pathophysiology of cardiovascular diseases [169,170]. Reduced levels of melatonin and its major metabolite, 6-sulphatoxymelatonin, have been reported in various cardiovascular diseases, including myocardial infarcts, coronary heart disease, congestive heart failure, and nocturnal hypertension [171,172]. Furthermore, melatonin deficiency caused by factors such as shift work, aging, and exposure to illuminated environments at night can result in glucose intolerance, insulin resistance, metabolic circadian disorganization, and sleep disturbance, all of which pose a threat to health conditions [173].

The timing of meals may also interact with melatonin to affect circadian rhythm and metabolism. Consuming a meal during nighttime when melatonin levels are elevated, particularly during night shifts, has been suggested as a potential mechanism for an elevated risk of heart disease and diabetes. A study conducted with 40 overweight/obese women of European ancestry who were habitual late eaters revealed that taking melatonin (5 mg) had a negative effect on glucose tolerance [174]. Specifically, the participants who had dinner within 2.5 h of their usual bedtime and had high levels of natural melatonin experienced a decrease in glucose tolerance. This suggests that when meal timing coincides with elevated melatonin levels, it impairs glucose tolerance. It is important to note that melatonin levels typically rise about 30 min before bedtime [175]. Another study utilized a mobile phone application with time-stamped pictures to track participants’ food intake over seven consecutive days, while also evaluating their body composition and the timing of melatonin release in a laboratory setting [14]. The findings revealed that individuals with higher body fat, known as non-lean individuals, consumed most of their calories approximately 1.1 h closer to the onset of melatonin release, which signifies the start of the biological night, compared with individuals with lower body fat, known as lean individuals [14]. These results provide additional evidence that the timing of meal intake throughout the circadian evening and/or night, independent of more conventional risk factors such as the quantity or content of consumed food and activity level, plays a vital role in determining body composition [14].

Therefore, in countries where dinner is served early, such as Sweden and Germany, the chances of food intake aligning with elevated melatonin levels are low [122]. However, in Spain, where dinner is usually around 10 p.m., melatonin levels at dinner time are approximately three times higher, especially among young individuals who have higher natural melatonin levels than older individuals [122]; this situation increases the likelihood of metabolic changes related to glucose [176]. In a randomized crossover study involving a Spanish population, researchers investigated the impact of late eating and elevated melatonin levels on glucose control, particularly in individuals carrying the G allele in the MTNR1B gene associated with type 2 diabetes [177]. The study found that late dinner timing resulted in significantly higher melatonin levels and impaired glucose tolerance, with lower insulin response and higher glucose levels. These effects were more pronounced in individuals carrying the G allele, suggesting that the combination of high melatonin and carbohydrate intake during late eating can lead to insulin secretion defects and impaired glucose control [177].

Therefore, it is advisable to have dinner early, around dusk, and refrain from consuming meals, particularly those with high glycemic content, in close proximity to exogenous melatonin intake or during nighttime when endogenous melatonin levels are typically elevated.

### 8.3. Other Mechanisms

In rodents, under the natural circadian setting, the light phase is associated with continuous suppression of feeding compared with the dark phase [178]. It has been consistently observed that food intake in normal rodents is significantly suppressed during the light phase compared with the dark phase [38,49]. This light-induced feeding inhibition is not simply due to the suppression of locomotor activity but is associated with the activation of anorexigenic neurons [179].

Oxytocin expression in the brain exhibits a circadian pattern synchrony with the light–dark cycle, with higher levels during the light phase in rodents [178,180,181,182]. Experimental evidence supports the role of nesfatin-1, derived from nucleobindin-2 (NUCB2) and expressed in the paraventricular nucleus (PVN), as a physiological anorexigenic peptide [178,183,184]. Nesfatin-1 attenuates food intake during the light phase in rodents through its action on oxytocin neurons in the PVN [178,183,185]. Light exposure, mediated via the SCN, activates the PVN oxytocin pathway, leading to the termination of feeding in mice [186]. The anorexigenic effect of oxytocin-induced suppression of food intake is related to nesfatin-1, as oxytocin administration increases the number of activated NUCB2/nesfatin-1 neurons in various brain regions, and this effect can be attenuated by inhibiting nesfatin-1 [187]. Altered levels of NUCB2/nesfatin-1 have been observed in obesity-related conditions in the rodent hypothalamus and human blood [178]. Furthermore, nesfatin-1, expressed in endocrine cells in the pancreas, has recently emerged as an important player in the regulation of glucose homeostasis through its insulinotropic action [178,187]. Based on these findings and the independent anorexigenic effect of nesfatin-1 from leptin, it is worth exploring its potential as a target for further research aimed at evaluating its efficacy as a treatment for obesity and type 2 diabetes mellitus [178,187,188].

Further research is required to investigate the link between nighttime light exposure in humans and the interplay among nesfatin-1, NUCB2, and oxytocin neurons in the paraventricular nucleus (PVN). Understanding this association can provide valuable insights into its impact on human appetite and metabolism

## 9. Meal Timing, Circadian Rhythm, and Gut Microbiota

There is a growing acknowledgment of the crucial role played by the gut microbiome in human health. Additionally, substantial variations in microbiome composition have been observed among different global lifestyles, and it is probable that the modern lifestyle has an impact on the gut microbiome [189]. In recent years, the connection between circadian rhythms and the composition of the gut microbiota (GM) has gained attention [190]. It has been observed that over 50% of the overall microbial composition exhibits rhythmic fluctuations throughout the day [190]. Studies have demonstrated that the gut microbiota, in both animal and human models, undergoes daily fluctuations influenced by feeding patterns, where the composition of the microbiota undergoes oscillations over 24 h, with 60% of the composition showing variation [191]. These fluctuations result in distinct compositional and functional profiles of the microbiota at different times of the day. Additional research conducted in mice validated these discoveries, demonstrating that even though gut microbes are not directly influenced by light, diurnal signals from the host organism trigger fluctuations in both the abundance and functional activity of the gut microbiota [192,193]. While the specific variations in different microbial species throughout the day are still an area of ongoing research, studies have shown that the overall makeup and functionality of the gut microbiota have the potential to exhibit diurnal fluctuations [191]. Research has demonstrated that certain microbial taxa, such as Bacteroidetes and Firmicutes, exhibit diurnal oscillations in their abundance, which may be influenced by factors like feeding/fasting cycles and the host’s internal circadian clock [194]. These oscillations in microbial abundance can impact the production of metabolites, including short-chain fatty acids (SCFAs), which play a role in host metabolism and health [192]. Furthermore, subsequent studies have highlighted the importance of time-specific functions in gut bacteria. For instance, the production of SCFAs, including butyrate, acetate, and propionate, by specific microbial species has been shown to exhibit diurnal patterns. SCFAs are known to influence host physiology, including energy metabolism, gut barrier function, and immune responses [195,196,197].

Moreover, data indicates that the variations in the gut microbiota are reliant on the proper functioning of the circadian clock gene network in the host organism [198,199].

Circadian disruption leads to various changes in gut function, such as increased gut permeability, which can cause dysfunction of the gut barrier and modify the composition of the gut microbiome [193,200]. Furthermore, research has revealed that the GM plays a role in synchronizing the host’s circadian biological clock through various signaling mechanisms [201].

The complex relationship between the circadian rhythm and the GM involves multiple communication pathways, forming a complex two-way system. Within this interplay, diet (composition and timing) plays a crucial role [190]. Therefore, any disruption or alteration in the circadian rhythm can significantly impact the rhythmicity of the gut microbiota, thereby leading to harmful effects on the host’s overall health [190]. Meal timing can have a major effect on the gut microbiota, both in animals and humans. The gut microbiota of mammals exhibits daily fluctuations regulated by the rhythmic patterns of food intake. The timing of food intake influences the daily fluctuations in the composition of the microbiota, and it is observed that the rhythmicity of the microbiota is a dynamic process that can be disrupted or restored in response to changes in feeding behaviors. Consequently, the timing of feeding serves as a connection between the circadian patterns in host behavior and the diurnal variations in the composition and role of the microbiota [191,202]. Furthermore, circadian misalignment in humans can modify the composition of the microbiota, potentially leading to an increase in proinflammatory taxa and a decrease in functional pathways mediated by the microbiota [203]. One of the affected pathways is the biosynthesis of tryptophan, which is crucial for serotonin production [203].

TRE has been revealed to reintroduce members of the Ruminococcaceae family, specifically within the Oscillibacter genus. These members are believed to confer resistance to the metabolic effects of obesity [191,202]. A higher abundance of Firmicutes species in the gut microbiome has been associated with increased adiposity, suggesting their potential involvement in obesity development [204]. However, research evaluating the microbiome in both normal mice and mice under TRE conditions indicates that the abundance of Firmicutes species is more closely linked to the food and feeding pattern rather than obesity or dysmetabolism per se [204]. One prevalent explanation for the documented advantages of TRE is that it emulates innate eating patterns aligned with circadian rhythms, resembling the dietary habits of humans prior to the advent of artificial lighting and the availability of high-energy foods around the clock [4,205,206].

During a study, male Wistar rats were subjected to a 16-h light and 8-h dark cycle. They were divided into four groups, each following specific dietary patterns that mimicked breakfast, lunch, dinner, and late-night eating [207]. Remarkably, engaging in late-night eating habits, such as skipping dinner for a night eating (BLN) or skipping breakfast and having a night eating (LDN), led to significant changes in the composition and functions of the gut microbiota [207]. These alterations in the gut microbiota are believed to be involved in the emergence of metabolic disorders.

A small cross-sectional study involving human adults with metabolic syndrome examined the associations between sleep quality, night eating behavior, and gut microbiome composition [208]. The study’s findings indicated potential links between the composition of the gut microbiome and disturbances in circadian rhythms caused by sleep disturbances or late-night eating; nevertheless, it was difficult to determine if the changes in microbiota are related to late eating, sleep disruption, or both. Additional evidence supporting the significance of meal timing in shaping the make-up of the gut microbiota was furnished by the findings of the initial investigation that examined the gut microbiota composition based on chronotype [209]. Carasso and colleagues discovered that individuals classified as evening chronotypes exhibited a greater abundance of Lachnospira [209]. This increased abundance was recently linked to the consumption of greater quantities of energy in the afternoon and evening, which is a typical characteristic of individuals classified as evening chronotypes [206]. Another small study revealed that individuals with late-night eating habits had a higher abundance of Erysipelotrichales, a member of the Firmicutes phylum [210]. This bacterial group was previously associated with metabolic disorders and obesity.

In summary, further research is needed to better understand the potential bidirectional relationship between meal timing and the gut microbiome and its implications for overall health. Future research needs to explore the relationship between mealtime, meal frequency and consistency, circadian rhythms, and GM; this area of research remains relatively unexplored thus far. Future studies should explore the specific mechanisms by which meal timing influences the rhythmicity and composition of the gut microbiota while controlling for other potential confounders like meal composition and sleep disruption. Also, investigations should aim to elucidate the bidirectional relationship between meal timing and the gut microbiota, including the potential role of specific bacterial taxa and functional pathways. Understanding these connections will provide valuable insights into the interplay between meal timing, circadian rhythms, and metabolic disorders.

## 10. Concluding Remarks and Future Directions

The role of diet in maintaining good health is crucial, and aligning food consumption with an individual’s internal circadian clock has been shown to support metabolic well-being. Innovative dietary approaches like time-restricted eating (TRE) have the potential to enhance circadian alignment, leading to a reduction in various metabolic risks [211,212,213]. However, despite the advantages of intermittent fasting and TRE for health, certain limitations exist. There is currently no consensus on the optimal timing of eating and fasting for achieving the best health outcomes, resulting in variations in meal timing across different studies. Moreover, one notable methodological limitation is that TRE inherently results in moderate calorie restriction, which makes it challenging to differentiate the effects of TRF from those of calorie restriction [29,214,215]. Meal timing significantly influences human physiology, and when there is a mismatch between feeding/fasting patterns and the endogenous circadian system, it can impact an individual’s health. The concept of meal timing presents a promising and innovative dietary strategy that holds importance for metabolic health.

The synchronization between the central and peripheral clocks is key for metabolic regulation. Disruptions in this synchronization, such as consuming meals late in the day, have been connected to an elevated susceptibility to metabolic disorders. On the other hand, early time-restricted eating, such as having dinner earlier in the evening, has been shown to positively influence blood glucose levels and substrate oxidation. Aligning meal timing with the natural circadian rhythm may yield favorable effects on metabolic health.

Late-night meals and going to bed soon after can disrupt the natural transition from the daytime feeding state to the nocturnal fasting state. This can result in prolonged increases in blood sugar levels. Regularly eating dinner late at night and going to bed soon after should prompt individuals to re-evaluate their late-night eating habits and overall lifestyle choices. Considering the impact on heart and metabolic health, it is essential to be mindful of the timing of our meals.

The precise mechanisms that underlie these associations are not yet fully comprehended. One limitation of large observational studies is the absence of direct measurements of the circadian system or circadian phase, which refers to the timing of the internal clock. Most of the previously described studies did not directly assess the internal circadian system, such as dim light melatonin onset or body temperature rhythms, thus making it challenging to evaluate the extent of circadian rhythm disruption.

Eating at inappropriate times can disrupt the alignment of circadian rhythms in different bodily tissues. Metabolites derived from food consumption also serve as time signals for peripheral clocks. Nevertheless, it is crucial to acknowledge that other factors, such as an unhealthy diet, low sleep quality or duration, and reduced physical activity, can also contribute to poor health. These noncircadian mechanisms may further connect these factors to negative health outcomes. Gaining a better understanding of the underlying mechanisms is essential in order to determine whether and how to intervene in relation to the behaviors above.

Circadian hormones, particularly cortisol, and melatonin, play pivotal roles in the regulation of metabolic processes. Cortisol, which follows a circadian pattern aligned with dawn in diurnal mammals, assists in the activation of energy reserves, appetite stimulation, and peripheral clock functioning. Glucocorticoids, including cortisol, influence the expression of clock genes and the synchronization of peripheral clocks with the central clock. Melatonin, responsible for regulating the sleep–wake cycle, also possesses antioxidant and anti-inflammatory properties. Meal timing that coincides with elevated melatonin levels, especially during the circadian evening and night, may impair glucose tolerance. Being mindful of melatonin release and avoiding meals close to bedtime or during elevated melatonin levels can help maintain healthy glucose control.

To advance our understanding of circadian health and metabolic well-being, additional studies are required to establish the optimal meal timing and dietary patterns. This research should involve direct measurements of the circadian system and account for noncircadian factors that affect metabolic risk, such as diet quality, sleep quality and duration, and physical activity levels. Furthermore, well-designed RCTs with adequate sample sizes and appropriate follow-up periods are necessary to understand the meal timing and dietary patterns that enhance circadian and metabolic health. Additionally, research should explore the relationship between mealtime, circadian rhythms, and gut microbiota.

## Figures and Tables

**Table 1:** A Summary of Randomized Controlled Trials Investigating the Relationship between Mealtimes, Circadian Rhythm, and Metabolism.

Authors (Year)	Study Design	Main Results
Nakamura K. et al., 2021 [100]	A randomized cross-over trial assessed the effect of early evening meals on blood glucose levels and postprandial lipid metabolism in healthy adults. Twelve participants (two males and ten females) completed a 3-day study, alternating between late (21:00) and early (18:00) dinners. Continuous blood glucose monitoring and metabolic measurements were conducted on day 3 using indirect calorimetry	Significant differences between the two groups were observed in mean 24 h blood glucose levels on day 2. There was a significant decrease in the postprandial respiratory quotient 30 min and 60 min after breakfast on day 3 in the early dinner group compared with the late dinner group.
Xie Z. et al., 2022 [104]	A randomized controlled trial compared two TRF regimens (early and midday) in healthy non-obese individuals. In total, 90 participants were randomized to eTRF (*n* = 30), mTRF (*n* = 30), or control groups (*n* = 30), and 82 participants completed the five-week trial and were analyzed (28 in eTRF, 26 in mTRF, 28 in control groups). Primary outcome: change in insulin resistance.	eTRF was more effective than mTRF at improving insulin sensitivity; eTRF, but not mTRF, improved fasting glucose, reduced total body mass and adiposity, ameliorated inflammation, and increased gut microbial diversity.
Bo S. et al., 2015 [96]	A randomized cross-over study assessed food-induced thermogenesis in morning and evening. Twenty subjects received the same standard meal in the morning and, 7 days later, in the evening (or vice versa). Calorimetry and blood sampling were performed at specific time intervals. General linear models were used to evaluate the “morning effect” compared to the evening effect.	Fasting resting metabolic rate (RMR) remained unchanged between morning and evening. After-meal RMR was significantly higher following the morning meal compared with the evening meal. RMR increased significantly after the morning meal. Glucose, insulin, and fatty acid concentrations showed delayed and larger increases after the evening meals.
Bandin C. et al., 2015 [95]	In a randomized cross-over trial, thirty-two women completed two randomized cross-over protocols: one protocol (P1) included an assessment of resting energy expenditure (indirect-calorimetry) and glucose tolerance (mixed-meal test) (*n* = 10) and the other (P2) included circadian-related measurements based on profiles in salivary cortisol and wrist temp. (T wrist) (*n* = 22). In each protocol, participants were provided with standardized meals during the two meal intervention weeks and were studied under two lunch-eating conditions: Early eating (EE; lunch at 01:00 p.m.) and late eating (LE; lunch at 04:30 p.m.).	LE, compared with EE, resulted in decreased pre-meal resting-energy expenditure, a lower pre-meal protein-corrected respiratory quotient (CRQ), and a changed post-meal profile of CRQ. These changes reflected a significantly lower pre-meal utilization of carbohydrates in LE versus EE. LE also increased glucose area under the curve above baseline by 46%, demonstrating decreased glucose tolerance. Changes in the daily profile of cortisol and T wrist were also found with LE blunting the cortisol profile, with lower morning and afternoon values, and suppressing the postprandial response.
Manoogian E.N.C. et al., 2022 [98]	In a randomized control trial including 137 firefighters who worked 24 h shifts (23–59 years old, 9% female), 12 weeks of 10 h time-restricted eating (TRE) was feasible, with TRE participants decreasing their eating window (baseline, mean 02:13 p.m., 95% CI 13.78–14.47 h; intervention, 11:13 a.m., 95% CI 10:73–11:54 h, *p* = 3.29 × 10^−17^).	Compared with the standard of care (SOC) arm, TRE significantly decreased VLDL particle size. In participants with elevated cardiometabolic risks at baseline, there were significant reductions in TRE compared with SOC in glycated hemoglobin A1C and diastolic blood pressure.
Qian J. et al., 2018 [102]	Using a randomized cross-over trial, the study aimed to discern the individual and combined effects of the circadian system and environmental/behavioral cycles, particularly circadian misalignment, on insulin sensitivity and β-cell functionality. This assessment was performed using the minimal oral model on 14 healthy individuals over two 8-day laboratory sessions. Each session started with 3 days under regular sleep/wake patterns. This was then followed by 4 days where participants either maintained their usual bedtime (indicating circadian alignment) or shifted to a 12-h inverted schedule, leading to circadian misalignment.	Data showed that the circadian phase and circadian misalignment affected glucose tolerance through different mechanisms. While the circadian system reduced glucose tolerance in the biological evening compared with the biological morning mainly by decreasing both dynamic and static β-cell responsivity, circadian misalignment reduced glucose tolerance mainly by lowering insulin sensitivity not by affecting β-cell function.
Collado M.C. et al., 2018 [97]	In a cross-over trial involving 10 healthy, young, normal-weight females, the researchers investigated the influence of meal timing on the human microbiota present in both saliva and fecal samples. Their goal was to see if consuming food later in the day affects the daily patterns of human salivary microbiota. To delve deeper into this, they analyzed the salivary microbiota from samples taken at four distinct intervals over a 24-h period, aiming to shed more light on the link between when one eats and potential metabolic changes in humans.	A significant diurnal rhythm in salivary diversity and relative bacterial abundance (i.e., TM7 and Fusobacteria) across both early and late eating conditions was found. Meal timing affected diurnal rhythms in a diversity of salivary microbiota toward an inverted rhythm between eating conditions, and eating late increased the number of putative pro-inflammatory taxa, showing a diurnal rhythm in the saliva.
Pizinger T. et al., 2018 [101]	Using a randomized control trial, the study aimed to assess how sleep and meal timings individually and collectively influenced insulin sensitivity (Si) in overweight individuals. The study enrolled six participants, comprising four men and two women, though one participant did not finish. The trial used a 4-phase inpatient cross-over design, which varied based on sleep schedules: either standard (Ns: from midnight to 8:00 a.m.) or delayed (Ls: from 3:30 a.m. to 11:30 a.m.). Meal timings also varied: either regular (Nm: at intervals of 1-, 5-, 11-, and 12.5-h post-waking) or delayed (Lm: at intervals of 4.5-, 8.5-, 14.5-, and 16-h post-waking). After three days in each phase, Si was evaluated using an insulin-modified frequently sampled intravenous glucose tolerance test at the designated breakfast time and a meal tolerance test at the designated lunchtime.	Mealtime influenced concentrations of glucose (*p* = 0.012) and insulin (*p* = 0.069) during the overnight hours. Average cortisol concentrations between 22:00 and 07:00 h tended to be affected by mealtime. Melatonin concentrations from the overnight sampling period showed no effect on mealtime.
Morris C.J. et al., 2016 [99]	Using a randomized cross-over study, the study aimed to test the hypothesis that the endogenous circadian system and circadian misalignment separately affect glucose tolerance in shift workers, both independently from behavioral cycle effects, including nine healthy subjects. The intervention included simulated night work comprised of 12 h inverted behavioral and environmental cycles (circadian misalignment) or simulated day work (circadian alignment). Postprandial glucose and insulin responses to identical meals given at 8:00 a.m. and 8:00 p.m. were measured in both protocols.	Circadian misalignment increased postprandial glucose by 5.6% independent of behavioral and circadian effects (*p* = 0.0042).
Sharma A. et al., 2017 [103]	Using a randomized control trial, the study aimed to determine the effect of rotational shift work on glucose metabolism. Using a randomized cross-over study design, 12 healthy nurses performing rotational shift work underwent an isotope-labeled mixed meal test during a simulated day shift and a simulated night shift, enabling simultaneous measurement of glucose flux and beta cell function using the oral minimal model.	Postprandial glycemic excursion was higher during the night shift. The time to peak insulin, C-peptide, and nadir glucagon suppression in response to meal ingestion was also delayed during the night shift. While insulin action did not differ between study days, the beta cell responsivity to glucose and disposition index were decreased during the night shift.
Vujovic N. et al., 2022 [107]	A randomized, controlled, cross-over trial with 18 subjects was used to determine the effects of late versus early eating while rigorously controlling for nutrient intake, physical activity, sleep, and light exposure. The parameters measured were subjective (hunger) and objective (hormones related to metabolism)	Late eating increased hunger and altered appetite-regulating hormones, increasing waketime and the 24 h ghrelin leptin ratio (*p* < 0.0001 and *p* = 0.006, respectively). Furthermore, late eating decreased waketime energy expenditure and 24 h core body temperature.
Jamshed H. et al., 2019 [65]	This study used a 4-day randomized crossover design to investigate the impact of time-restricted feeding (TRF) on gene expression, circulating hormones, and diurnal patterns in cardiometabolic risk factors. Eleven overweight adults participated in the study, following two different eating schedules: early TRF (eTRF) from 8 a.m. to 2 p.m. and a control schedule from 8 a.m. to 8 p.m. Continuous glucose monitoring was conducted, and blood samples were collected to assess various factors.	eTRF resulted in improved glucose levels and glycemic excursions compared with the control schedule. In the morning, eTRF increased ketones, cholesterol, and the expression of stress response and aging gene SIRT1, as well as the autophagy gene LC3A. In the evening, eTRF tended to increase brain-derived neurotropic factor (BDNF) and significantly increased the expression of MTOR, a protein involved in nutrient sensing and cell growth. Additionally, eTRF altered diurnal patterns in cortisol levels and the expression of circadian clock genes.
Lowe D.A. et al., 2020 [108]	In this 12-week randomized clinical trial, participants (*n* = 116) were divided into two groups: the consistent meal timing (CMT) group, instructed to consume three structured meals per day, and the time-restricted eating (TRE) group, instructed to eat ad libitum from 12:00 p.m. until 8:00 p.m. The study aimed to investigate the impact of 16:8 h time-restricted eating on weight loss and metabolic risk markers. The study utilized a custom mobile study application, with in-person testing for a subset of 50 participants.	The TRE group had significant weight loss compared with the CMT group. The TRE group also had significant weight loss within the in-person cohort. Furthermore, the two groups showed a significant difference in appendicular lean mass index. No significant changes were observed in other secondary outcomes within or between the groups. Estimated energy intake did not differ significantly between the groups.
Hutchison A.T. et al., 2019 [105]	In this randomized controlled trial, the impact of 9 h TRF on glucose tolerance in men at risk for type 2 diabetes was assessed. Fifteen male middle-aged, obese participants wore a continuous glucose monitor for 7 days during the baseline assessment and two 7-day TRF conditions. They were randomly assigned to either early TRF (TRFe) from 8 a.m. to 5 p.m. or delayed TRF (TRFd) from 12 p.m. to 9 p.m., with a 2-week washout phase between conditions. Glucose, insulin, triglycerides, nonesterified fatty acids, and gastrointestinal hormone levels were measured and analyzed.	The results demonstrated that both TRFe and TRFd improved glucose tolerance, as evidenced by a reduction in glucose incremental area under the curve and fasting triglycerides (*p* = 0.003) on day 7 compared with day 0. However, no significant interactions between mealtime and TRF existed for any of the variables examined. TRF did not significantly affect fasting or postprandial insulin, nonesterified fatty acids, or gastrointestinal hormone levels. As measured using continuous glucose monitoring, mean fasting glucose was lower in TRFe but not in TRFd compared to baseline, with no significant difference observed between the two TRF conditions.
Jones R. et al., 2020 [106]	This randomized controlled trial investigated the chronic effects of early TRF (eTRF) compared to an energy-matched control on insulin and anabolic sensitivity in healthy males. In total, 16 young, lean participants were assigned to eTRF (*n* = 8) or control/caloric restriction (CON:CR; *n* = 8) groups. The eTRF group followed the eTRF diet for 2 weeks, restricting daily energy intake to the period between 08:00 and 16:00. The CON:CR group underwent a calorie-matched control diet after the eTRF intervention. Metabolic responses were assessed before and after the interventions, following a 12 h overnight fast, using a carbohydrate/protein drink.	The results showed that eTRF improved whole-body insulin sensitivity compared with CON:CR, with a between-group difference of 1.89. eTRF also enhanced skeletal muscle uptake of glucose (between-group difference: 4266 μmol·min^−1^·kg^−1^·180 min; 95% CI: 261, 8270; *p* = 0.04; η2p = 0.31) and branched-chain amino acids (BCAAs). The eTRF group experienced a reduction in energy intake (approximately 400 kcal·d^−1^) and weight loss, which was comparable to the weight loss observed in the CON:CR group
Blum et al., 2023 [109]	In a trial with 15 adults who typically slept late, the participants were randomly assigned to follow either early time-restricted eating (eTRE) practices or a general sleep and nutrition regimen, both introduced using a video session. Sleep patterns were monitored over three weeks, encompassing an initial baseline week and a two-week intervention phase.	Those following early eTRE began their sleep cycle earlier and woke up sooner than those in the control group. Although eTRE participants showed a minor uptick in sleep duration, the change was not notably significant. The results suggest eTRE’s potential in adjusting late sleep habits.

Abbreviations: eTRF and mTRF, early and midday time-restricted feeding; RMR: resting metabolic rate; T wrist: wrist temperature; EE and LE: early eating and late eating, respectively.

## Data Availability

Not applicable.
